# Supplementary material for: Location and mapping of the human rostromedial tegmental nucleus and associated midbrain inhibitory nuclei regulating dopamine neurons
Source: Brain Struct Funct. 2026 May 13;231(5):64. doi: 10.1007/s00429-026-03103-x (PMC13171687; doi:10.1007/s00429-026-03103-x)
Supplement: Supplementary file 1 — Supplementary Material 1 [file 429_2026_3103_MOESM1_ESM.pdf]

Supplementary information

Location and mapping of the human rostromedial tegmental nucleus and associated midbrain inhibitory nuclei regulating dopamine neurons

Anastasia Filimontseva[1,2], ORCID 0000-0002-3316-9035 email afil9180@uni.sydney.edu.au

YuHong Fu[1,2], ORCID 0000-0003-4539-2039 email yuhong.fu@sydney.edu.au

Glenda M Halliday[1,2,#] ORCID 0000-0003-0422-8398 email glenda.halliday@sydney.edu.au

[1]The University of Sydney Brain and Mind Centre & Faculty of Medicine and Health School of Medical Sciences, Sydney, NSW 2050, Australia

[2]Aligning Science Across Parkinson's (ASAP) Collaborative Research Network, Chevy Chase, MD, 20815

[#]Corresponding author: Professor Glenda Halliday, University of Sydney Brain and Mind Centre, 97 Church Street, Camperdown, NSW 2050 AUSTRALIA, Email: glenda.halliday@sydney.edu.au, Tel: +61 2 9351 0888

Supplementary Methods

Semi-automated QuPath quantification for cell typing

Morphological features of GABA and Nissl positive neurons were assessed using QuPath (ver.0.5.1, RRID:SCR_018257; https://qupath.github.io/). A semi-automated quantification method was devised to assess morphological features, cellular density and cell-type proportions in GABAergic cell clusters.

1. GABA neuronal area and circularity

GABA positive neurons in immunohistochemically processed GAD67 and TH stained sections (33.5mm – 38mm, Figure 2) were quantified with QuPath. Three separate 'pixel classifiers' were created for this analysis for each ROI - the interpeduncular nucleus (IPN), rostromedial tegmental nucleus (RMTg) and lateral cluster in the RRF (LatC). Positive

neuronal staining was manually traced in ROIs in several sections. These traced images were used to ‘train a pixel classifier’ for each ROI. In some instances, training images were modified to increase the suitability of a pixel classifier. Once a pixel classifier showed suitable live prediction output, a size threshold was set. For GABAergic neurons this was set to a minimum 250μm^2 and applied across all clusters.

Our GABA antibody (GAD67, RRID:AB_627650, Cat# sc-28376) abundantly labelled GABA positive neurons, but also labelled fibers and terminals. Some cases had more intense fiber and terminal GABA labelling, which challenged the neuronal-GABA pixel classifier. As a result, the neuronal-GABA pixel classifier output was manually assessed for every case. In sections where the classifier was not sufficient, GABA-positive cells were traced manually to assess cellular morphology in these clusters. Relevant cellular morphology features were extracted for further statistical testing.

2. Nissl neuronal area and circularity

CV-stained, serial sections from relevant Obex levels (33mm – 38mm, Figure 3) were analysed in QuPath using the same methods in the three ROIs. When the pixel classifier for Nissl stained neurons with a nucleolus showed suitable live prediction output, the size threshold was set. For Nissl stained neurons this was set to a minimum of 70μm^2. This size threshold was consistent for all three ROIs and was selected to limit identification of non-neuronal Nissl positive nuclei. These classifiers were run on the relevant ROIs in all sections analysed. Relevant cellular morphology features were extracted for further statistical testing.

3. Neuronal cell-typing analysis

Similar pixel classifier analysis was used to investigate GABA and non-GABA neuronal populations in sections stained immunohistochemically for GAD67 and TH and then CV. After de-coverslipping and re-hydration, the sections GAD67 and TH stained sections were counterstained with 0.5% CV (HB5608 Hello Bio) to analyse the non-GABA neuronal proportion. Using the same pixel classification protocol, appropriate classifiers were created, visually validated and applied to the clusters of interest. The minimum size threshold for neuronal Nissl was set at 50μm^2 for all regions in all sections. After extracting the total number of non-GABA neurons, we quantified total neuronal population in these regions by adding the previously quantified GABA-positive neurons from the same sections. We then performed statistical analysis for proportion and density of GABA and non-GABA cell types.

Supplementary Tables 1 and 2

Supplementary Table 1. Case details

Brain Bank	ID	Age	Sex[#]	ABC scores[*]	Analysis
Sydney Brain Bank, Australia	MJFF Case 3	85	F	2,0,1	GABA neuronal morphology and density, chemoarchitectural mapping and neuronal cell-typing analysis.
Sydney Brain Bank, Australia	MJFF Case 5	88	F	2,1,3	GABA neuronal morphology and density, chemoarchitectural mapping and neuronal cell-typing analysis.
Sydney Brain Bank, Australia	MJFF Case 11	91	F	1,2,0	GABA neuronal morphology and density, chemoarchitectural mapping and neuronal cell-typing analysis.
Sydney Brain Bank, Australia	MJFF Case 12	93	F	1,1,0	GABA neuronal morphology and density, chemoarchitectural mapping and neuronal cell-typing analysis.
Sydney Brain Bank, Australia	MJFF Case 7	97	M	1,1,0	Chemoarchitectural mapping.
Sydney Brain Bank, Australia	MJFF Case 8	100	F	0,1,0	Chemoarchitectural mapping.
Sydney Brain Bank, Australia	MJFF Case 9	87	M	2,1,0	Chemoarchitectural mapping.
Sydney Brain Bank, Australia	MJFF Case 10	76	F	0,1,0	Chemoarchitectural mapping.
Sydney Brain Bank, Australia	MJFF Case 4	93	M	2,0,1	GABA neuronal morphology and density, chemoarchitectural mapping, neuronal cell-typing and supplementary figure 4 cellular cluster identification.
Sydney Brain Bank, Australia	MJFF Case 6	93	F	2,1,1	GABA neuronal morphology and density, chemoarchitectural mapping and neuronal cell-typing analysis.
Flinders Medical Centre and Institute of Medical and Veterinary Sciences in South Australia	FMC Control 1	76	F	0,0,0	Nissl cellular morphology and cytoarchitectural mapping.
Flinders Medical Centre and Institute of Medical and Veterinary Sciences in South Australia	FMC Control 2	58	M	0,0,0	Nissl cellular morphology and cytoarchitectural mapping.
Flinders Medical Centre and Institute of Medical and Veterinary Sciences in South Australia	FMC Control 3	88	F	0,1,0	Nissl cellular morphology and cytoarchitectural mapping.
Flinders Medical Centre and Institute of Medical and Veterinary Sciences in South Australia	FMC Control 4	64	M	0,0,0	Nissl cellular morphology and cytoarchitectural mapping.

[#]M=male, F=female; *(Hyman et al. 2012)

Supplementary Table 2. Rostromedial tegmental nucleus (RMTg) identification in animal studies to identify relevant landmarks.

**Species**	**Rostral landmarks**	**Intermediate landmarks**	**Caudal landmarks**	**Method**	**Selective RMTg labeling**	**Reference**
Rat		CLi, IPN, xscp		Retrograde injections in VTA and lateral habenula to label RMTg neurons	Yes	(Schoukroun et al. 2024)
Rat	ml, IPN	IPN, scp	xscp	FosB and GABA double labeling after chronic cocaine administration	Yes	(Perrotti et al. 2005)
Rat	NA	NA	NA	VTA-injected retrogradely labeled RMTg neurons co-expressing GAD67; and retrogradely labeled Fos positive neurons	Yes	(Jhou et al. 2009a)
Rat	Posterior VTA	NA	Xscp, cp, ml, IPN, tth	FosB immunoreactivitiy with psychostimulant injections	Yes	(Kaufling et al. 2010)
Rat	Posterior VTA and PN, ml, IPN, CLi, RRF, RN, cp	xscp, CLi, IPN, ml, tth, RRF, cp	xscp, CLi, IPN, Pn, cp, ml, tth, RRF	Double immunostaining for FosB and GABA	Yes	(Kaufling et al. 2009)
Rat	CLi, cp, RRF, IPN	NA	NA	Klüver-Barrera stain	No	(Petzel et al. 2017)
Mouse	NA	NA	NA	Anterograde injection into lateral habenula and GAD67 immunofluorescence	No	(Lammel et al. 2012)
Mouse	NA	NA	NA	Sox14 and FoxP1 *in-situ* hybridization and immunohistochemical labeling in embryonic mice	Yes	(Lahti et al. 2016)
Mouse	IPN, VTA, RLi	MnR, CLi	MnR	Viral injections encoding muscarinic Gq protein-coupled receptors (hM3Dq) injected into RMTg	Yes	(Zhao et al. 2022)
Non-human primates		xscp, ml, mlf, IPN, NRTP		Electrophysiological recordings of orthodromic and antidromic stimulations of lateral habenula and SNpc. Recorded RMTg neurons were histologically reconstructed. RMTg boundaries were delineated with retrograde tracing.		(Hong et al. 2011)

CLi=caudal linear nucleus, cp=cerebral peduncle, IPN=interpeduncular nucleus, ml=medial lemniscus, mlf=medial ongitudinal fasciulus, MnR=median raphe nucleus, NRTP=nucleus reticularis tegmenti pontis , Pn=pontine nuclei, PN=paranigral nucleus, RLi=rostral linear nucleus, RN=red nucleus, RRF=retrorubral fields, scp=superior cerebellar peduncle, SNpc=substantia nigra pars compacta, tth=trigeminothalamic tract, VTA=ventral tegmental area, xscp=decussation of the scp

Supplementary Figures 1-3

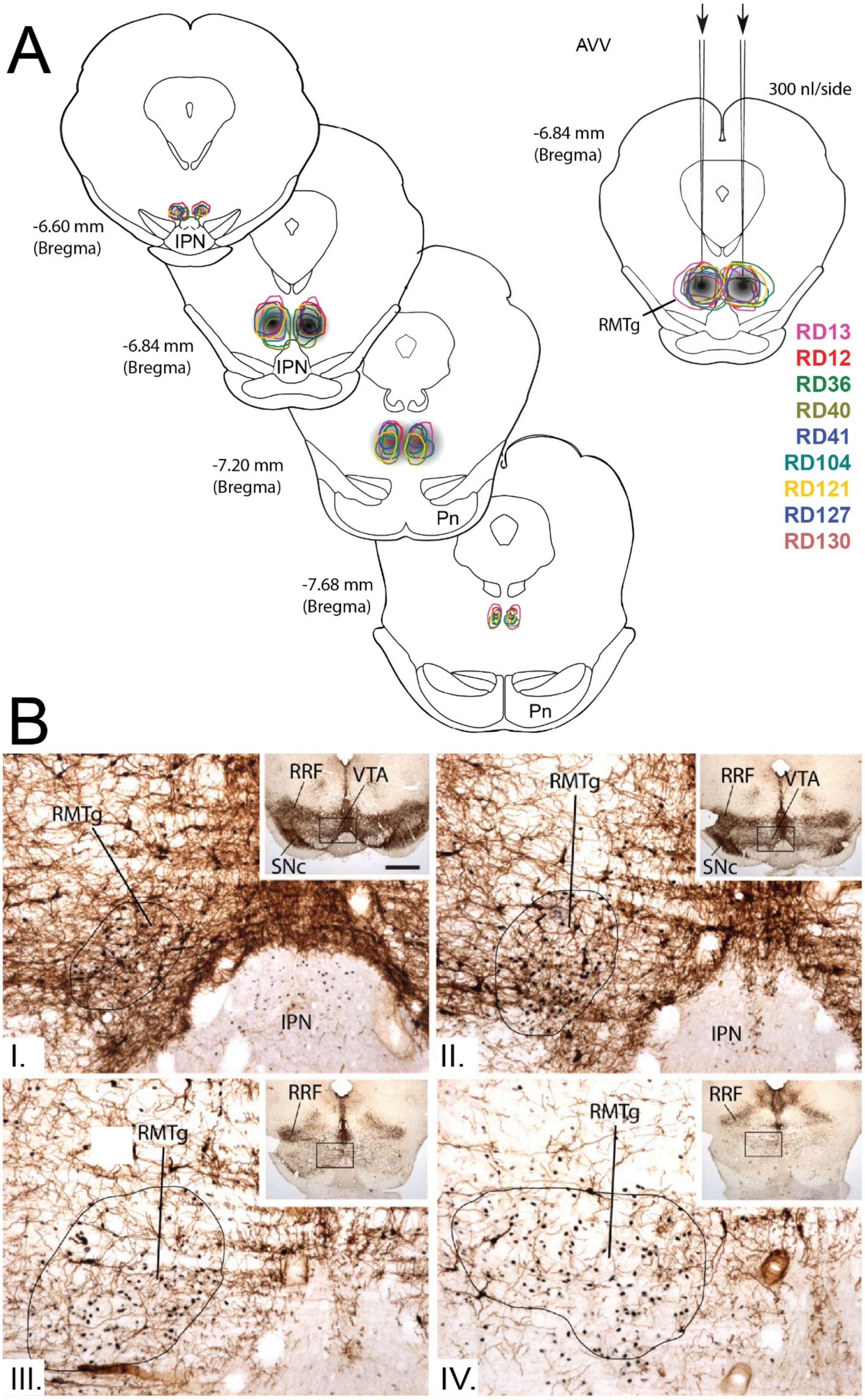

Supplementary Figure 1. ***Identification and mapping of rostromedial tegmental nucleus (RMTg) in rodent using stereotaxic surgery and immunohistochemistry.*** **A)** Adeno-associated virus (AAV) vectors expressing hM3Dq receptors targeted RMTg at different rostro-caudal levels in nine rats. Figure adapted with permission from *Yang et al., 2018* (Yang et al. 2018). **B)** RMTg neurons also express Fos protein (black reaction product) after methamphetamine injection. Fos-labeled neurons are embedded in tyrosine hydroylase (TH) immunoreactive processes and cell bodies (brown) in more rostral sections, I) and II). There is less TH immunoreactivity in caudal sections, III) and IV). The RMTg outline is larger in more caudal sections. Figure adapted with permission from *Jhou et al., 2009* (Jhou et al. 2009b). Abbreviations: aqueduct (Aq); pontine nuclei (Pn); retrorubral field (RRF); interpeduncular nucleus (IPN); substantia nigra pars compacta (SNc); ventral tegmental area (VTA).

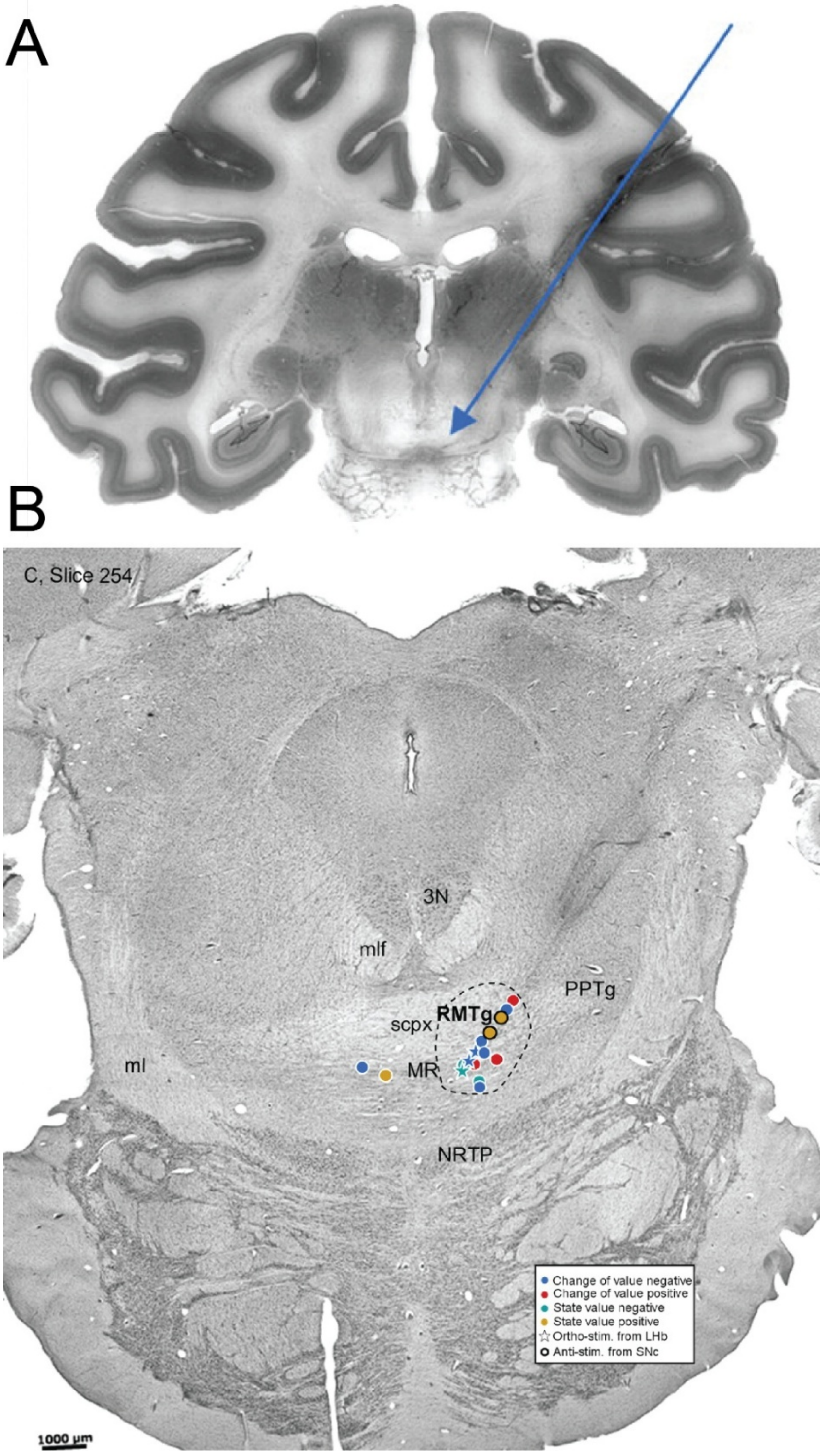

Supplementary Figure 2. ***Identification of rostromedial tegmental nucleus (RMTg) in rhesus monkeys.*** **A)** Electrode tract schematic to identify RMTg neurons during a reward-related visual task. **B)** Identity of recorded neurons was confirmed as RMTg using orthodromic and antidromic stimulations from lateral habenula and substantia nigra pars compacta (SNpc), respectively. Estimated locations of recorded neurons are histologically mapped. Figure adapted with permission from *Hong et al., 2011* (Hong et al. 2011). Abbreviations: oculomotor nucleus (3N); medial lemniscus (ml); medial longitudinal fascilicus (mlf); median raphe (MR); pedunculopontine tegmental nucleus (PPTg); superior cerebellar peduncle decussation (scpx); nucleus reticularis tegmenti pontis (NRTP).

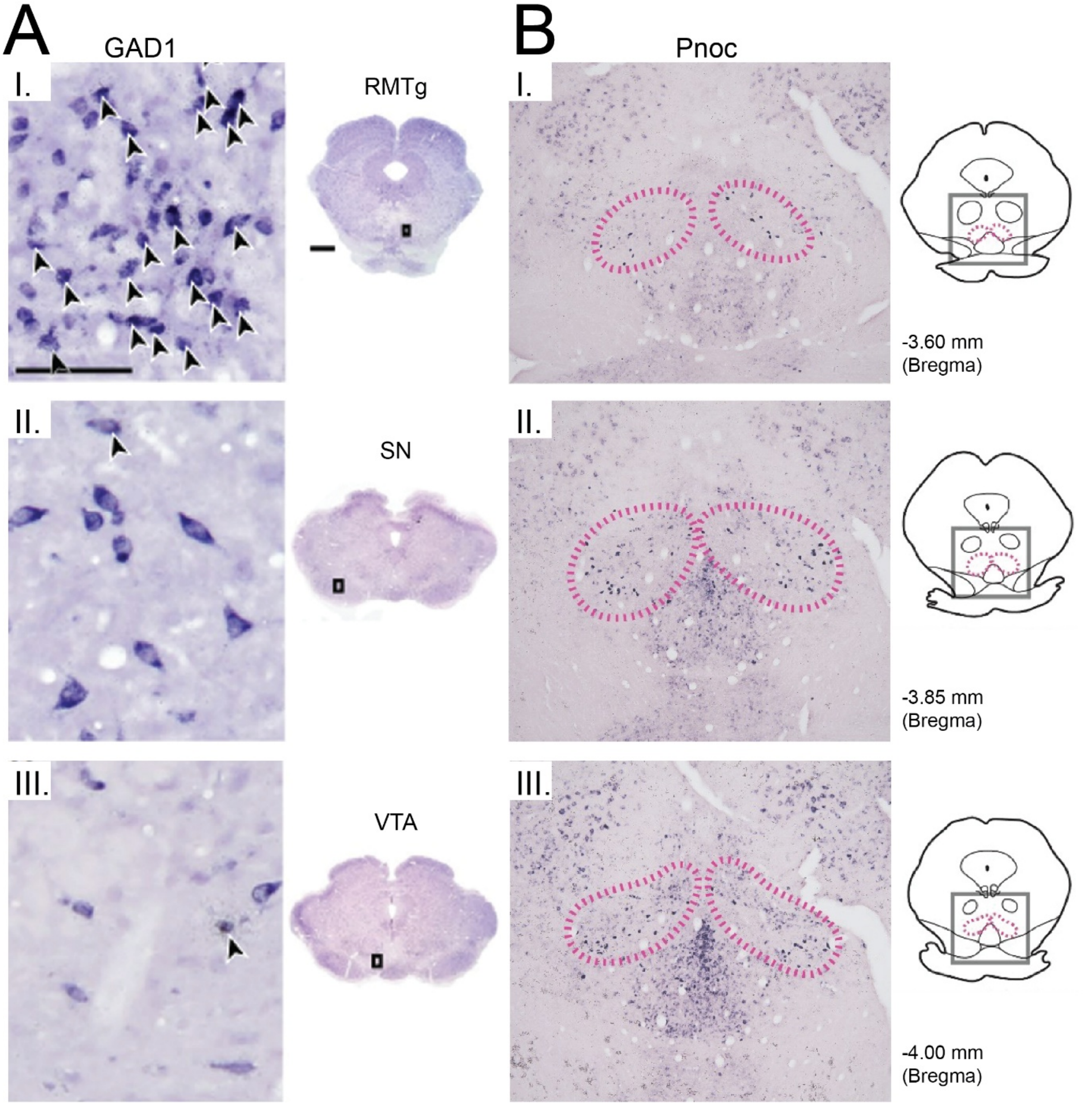

Supplementary Figure 3. ***Comparison of rostromedial tegmental nucleus (RMTg) neurons to neighbouring structures using in-situ hybridization.*** **A)** Inhibitory neurons in RMTg, substantia nigra (SN) and ventral tegmental area (VTA) are labelled with *GAD1 in-situ* hybridization. RMTg neurons are smaller and more dense in I), compared to SN and VTA neurons in II) and III). **B)** RMTg neurons also appear more dense and larger than neighbouring interpeduncular nucleus (IPN) when labeled with *in-situ* hybridization for *Pnoc* mRNA. Figure adapted permission from *Smith et al., 2019* (Smith et al. 2019)

Supplementary References

Hong S, Jhou TC, Smith M, Saleem KS, Hikosaka O (2011) Negative Reward Signals from the Lateral Habenula to Dopamine Neurons Are Mediated by Rostromedial Tegmental Nucleus in Primates. The Journal of Neuroscience 31 (32):11457-11471. doi:10.1523/jneurosci.1384-11.2011

Hyman BT, Phelps CH, Beach TG, Bigio EH, Cairns NJ, Carrillo MC, Dickson DW, Duyckaerts C, Frosch MP, Masliah E, Mirra SS, Nelson PT, Schneider JA, Thal DR, Thies B, Trojanowski JQ, Vinters HV, Montine TJ (2012) National Institute on Aging-Alzheimer's Association guidelines for the neuropathologic assessment of Alzheimer's disease. Alzheimers Dement 8 (1):1-13. doi:10.1016/j.jalz.2011.10.007

Jhou TC, Fields HL, Baxter MG, Saper CB, Holland PC (2009a) The Rostromedial Tegmental Nucleus (RMTg), a GABAergic Afferent to Midbrain Dopamine Neurons, Encodes Aversive Stimuli and Inhibits Motor Responses. Neuron 61 (5):786-800. doi:https://doi.org/10.1016/j.neuron.2009.02.001

Jhou TC, Geisler S, Marinelli M, Degarmo BA, Zahm DS (2009b) The mesopontine rostromedial tegmental nucleus: A structure targeted by the lateral habenula that projects to the ventral tegmental area of Tsai and substantia nigra compacta. J Comp Neurol 513 (6):566-596. doi:10.1002/cne.21891

Kaufling J, Veinante P, Pawlowski SA, Freund-Mercier MJ, Barrot M (2009) Afferents to the GABAergic tail of the ventral tegmental area in the rat. J Comp Neurol 513 (6):597-621. doi:10.1002/cne.21983

Kaufling J, Waltisperger E, Bourdy R, Valera A, Veinante P, Freund-Mercier M-J, Barrot M (2010) Pharmacological recruitment of the GABAergic tail of the ventral tegmental area by acute drug exposure. British Journal of Pharmacology 161 (8):1677-1691. doi:https://doi.org/10.1111/j.1476-5381.2010.00984.x

Lahti L, Haugas M, Tikker L, Airavaara M, Voutilainen MH, Anttila J, Kumar S, Inkinen C, Salminen M, Partanen J (2016) Differentiation and molecular heterogeneity of inhibitory and excitatory neurons associated with midbrain dopaminergic nuclei. Development 143 (3):516-529. doi:10.1242/dev.129957

Lammel S, Lim BK, Ran C, Huang KW, Betley MJ, Tye KM, Deisseroth K, Malenka RC (2012) Input-specific control of reward and aversion in the ventral tegmental area. Nature 491 (7423):212-217. doi:10.1038/nature11527

Perrotti LI, Bolaños CA, Choi K-H, Russo SJ, Edwards S, Ulery PG, Wallace DL, Self DW, Nestler EJ, Barrot M (2005) ΔFosB accumulates in a GABAergic cell population in the posterior tail of the ventral tegmental area after psychostimulant treatment. European Journal of Neuroscience 21 (10):2817-2824. doi:https://doi.org/10.1111/j.1460-9568.2005.04110.x

Petzel A, Bernard R, Poller WC, Veh RW (2017) Anterior and posterior parts of the rat ventral tegmental area and the rostromedial tegmental nucleus receive topographically distinct afferents from the lateral habenular complex. J Comp Neurol 525 (10):2310-2327. doi:10.1002/cne.24200

Schoukroun F, Befort K, Bourdy R (2024) The rostromedial tegmental nucleus gates fat overconsumption through ventral tegmental area output in male rats. Neuropsychopharmacology 49 (10):1569-1579. doi:10.1038/s41386-024-01855-w

Smith RJ, Vento PJ, Chao YS, Good CH, Jhou TC (2019) Gene expression and neurochemical characterization of the rostromedial tegmental nucleus (RMTg) in rats and mice. Brain Structure and Function 224 (1):219-238. doi:10.1007/s00429-018-1761-7

Yang S-R, Hu Z-Z, Luo Y-J, Zhao Y-N, Sun H-X, Yin D, Wang C-Y, Yan Y-D, Wang D-R, Yuan X-S, Ye C-B, Guo W, Qu W-M, Cherasse Y, Lazarus M, Ding Y-Q, Huang Z-L (2018) The rostromedial tegmental nucleus is essential for non-rapid eye movement sleep. PLOS Biology 16 (4):e2002909. doi:10.1371/journal.pbio.2002909

Zhao Y-N, Jiang J-B, Tao S-Y, Zhang Y, Chen Z-K, Qu W-M, Huang Z-L, Yang S-R (2022) GABAergic neurons in the rostromedial tegmental nucleus are essential for rapid eye movement sleep suppression. Nature Communications 13 (1):7552. doi:10.1038/s41467-022-35299-x
